# Characterization of the Bacterial Community Naturally Present on Commercially Grown Basil Leaves: Evaluation of Sample Preparation Prior to Culture-Independent Techniques

**DOI:** 10.3390/ijerph120810171

**Published:** 2015-08-21

**Authors:** Siele Ceuppens, Stefanie Delbeke, Dieter De Coninck, Jolien Boussemaere, Nico Boon, Mieke Uyttendaele

**Affiliations:** 1 Faculty of Bioscience Engineering, Department of Food Safety and Food Quality, Laboratory of Food Microbiology and Food Preservation (LFMFP), Ghent University, Ghent 9000, Belgium; E-Mails: siele.ceuppens@ugent.be (S.C.); stefanie_dlbk@hotmail.com (S.D.); jolien_boussemaere@hotmail.com (J.B.); 2 Faculty of Pharmaceutical Sciences, Department of Pharmaceutics, Laboratory of Pharmaceutical Biotechnology (LabFBT), Ghent University, Ghent 9000, Belgium; E-Mail: dieter.deconinck@ugent.be; 3 Faculty of Bioscience Engineering, Department of Biochemical and Microbial Technology, Laboratory of Microbial Ecology and Technology (LabMET), Ghent University, Ghent 9000, Belgium; E-Mail: nico.boon@ugent.be

**Keywords:** *Novosphingobium*, fresh herbs, 16S rRNA, DGGE, next-generation sequencing NGS

## Abstract

Fresh herbs such as basil constitute an important food commodity worldwide. Basil provides considerable culinary and health benefits, but has also been implicated in foodborne illnesses. The naturally occurring bacterial community on basil leaves is currently unknown, so the epiphytic bacterial community was investigated using the culture-independent techniques denaturing gradient gel electrophoresis (DGGE) and next-generation sequencing (NGS). Sample preparation had a major influence on the results from DGGE and NGS: *Novosphingobium* was the dominant genus for three different basil batches obtained by maceration of basil leaves, while washing of the leaves yielded lower numbers but more variable dominant bacterial genera including *Klebsiella, Pantoea, Flavobacterium, Sphingobacterium* and *Pseudomonas*. During storage of basil, bacterial growth and shifts in the bacterial community were observed with DGGE and NGS. Spoilage was not associated with specific bacterial groups and presumably caused by physiological tissue deterioration and visual defects, rather than by bacterial growth.

## 1. Introduction

The microbiota of the phyllosphere, *i.e.*, all plant surfaces above the ground, is dominated by bacteria over Archaea and fungi, typically reaching bacterial densities of 6 to 7 log cells per cm² leaf [1]. The conditions on the plant leaves are harsh for bacteria, with large temporal (day–night fluctuation, seasonal differences) and spatial variations in solar irradiation and the availability of water and nutrients, resulting in local microsites at which conditions are favorable for growth and/or survival [2,3,4,5]. Indigenous epiphytic bacteria may display various beneficial effects for the plant in terms of promoting growth and/or health, e.g., phytohormone production, improvement of the availability and/or uptake of nutrients and inhibition of plant pathogens by biosynthesis of antimicrobial compounds and induction of systemic resistance [1]. Moreover, the indigenous microbiota influences the survival and persistence of human pathogens, for example *Erwinia* spp. and *Pseudomonas* spp. inhibited *Escherichia coli* O157:H7 on spinach leaves [6]. On the other hand, positive interactions between pathogens and the native microbiota on plants also exist, for example the survival of *Salmonella* on produce is increased by soft rot bacteria such as *Erwinia carotovora* and *Pseudomonas viridiflava* [7], probably due to the increased availability of nutrients. 

Fresh herbs are an important component in the contemporary cooking and consumption patterns [8]. The use of fresh herbs has been reported by 91 % of Belgian and 73 % of Spanish respondents, at least monthly or more frequently for 92 % of these consumers [9]. In Norway, 60 % of the consumers ate fresh basil, with average portions of approximately one gram [10]. Supermarkets offer a wide choice of fresh herbs throughout the year, either as whole plants or as ready-to-use cut leaves in plastic trays. Leafy greens in their natural state are susceptible to spoilage by micro-organisms, in particular cut leaves, because the intact cell structure provides a protective barrier that is damaged by processing [11]. The overall microbial quality of foods of leafy greens such as fresh herbs are still often assessed by using total mesophilic aerobic plate counts, although it has been acknowledged that this is not a reliable indicator to judge neither the sanitary quality nor the sensorial quality of leafy greens [12]. It is of interest to have better knowledge about the composition of the natural indigenous microbiota and its changes in time during storage to get insight on the dominant spoilage microbiota. Therefore, more knowledge about the bacteria which are naturally present on basil leaves (*i.e.*, the epiphytic bacteria) is warranted, because characterization and understanding of the bacterial community and ecology during storage of basil leaves will facilitate the understanding of which microbial groups should be targeted in assessing microbial quality.

Conventional culture methods detect only a minority (max. 3 %) of the bacteria in environmental samples (water, soil, sediment and sludge) [13,14]. Bacterial communities in environmental and food samples have already been extensively investigated by culture-independent techniques such as denaturing gradient gel electrophoresis (DGGE) to avoid the culturing bias while studying microbial fermentations in food and community dynamics [15,16,17,18,19,20]. Moreover, DGGE is very useful to rapidly check the impact of a culturing step on the diversity of the bacterial community. DGGE patterns reflect the total composition and diversity in the sample, but identification of the species behind specific bands requires cutting, purifying, cloning and sequencing of the band(s). Instead of this laborious procedure, next-generation sequencing (NGS) of the 16S rRNA gene can be applied for taxonomic identification of all bacteria present in the sample. NGS is increasingly applied as an alternative molecular technique in food and clinical microbiology [21,22,23,24,25,26]. NGS has the advantages of short analysis time, high specificity and high resolution. 

Given the increasing importance of fresh herbs in the contemporary consumption patterns, the bacterial community on basil leaves was studied. No studies are currently available about the total bacterial community on basil without prior enrichment or cultivation steps which are known to create a significant culture bias. Therefore, the total bacterial community on basil leaves and changes of this community during storage and spoilage of cut basil leaves at different temperatures (7 °C, 15 °C and 22 °C) were investigated by culture-independent techniques denaturing gradient gel electrophoresis (DGGE) and next-generation sequencing (NGS). Special attention was given to evaluation of the sample preparation methods.

## 2. Experimental Section 

### 2.1. Basil

**Figure 1:**
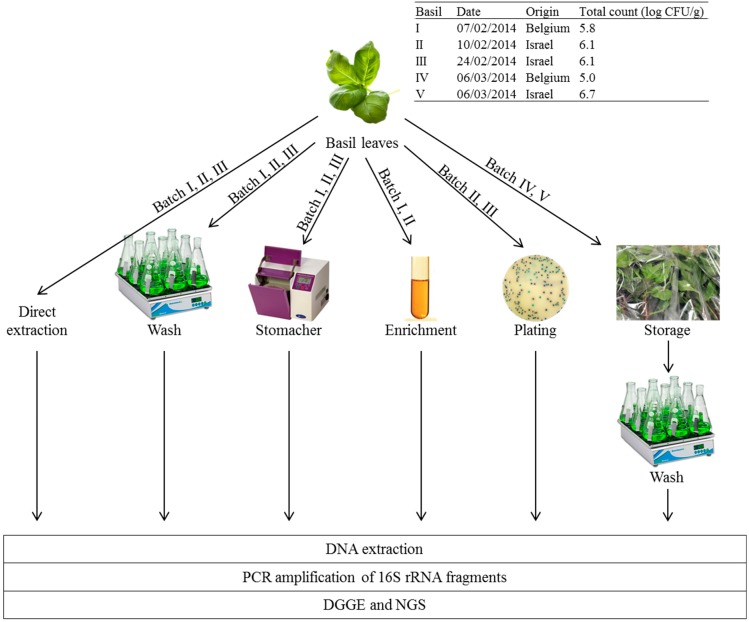
Overview of the five basil batches used and the experimental set-up of this study to characterization of the epiphytic bacterial community of basil by molecular techniques denaturing gradient gel electrophoresis (DGGE) and next-generation sequencing (NGS), both targeting the 16S rRNA gene.

To capture the existing variability on the Belgian retail market for basil, both basil leaves imported from Israel in plastic trays as well as whole basil plants from Belgian organic culture, from which the leaves were removed in the laboratory. Figure 1 presents an overview of the different batches of basil leaves which were analyzed in this study.

### 2.2. Molecular Microbiological Analyses

#### 2.2.1. Sample Preparation

Three different sample preparation methods were tested on basil batch I, II and III prior to DNA extraction as described below in Section 2.2.2. (Figure 1). Direct extraction of the total microbial DNA was done with approx. 300 mg basil leave (directly mixed with 1 mL extraction buffer or 2 mL lysis buffer). Maceration of 20 to 30 g of basil leaves (corresponding with all full-grown leaves of a retail plant) was done in 200 to 300 mL PPS (a tenfold dilution) for 1 min in a stomacher (L.E.D. Techno) laboratory blender. Washing of 20 to 30 g of basil leaves was done in 400 to 600 mL PPS with 1 % Tween 80^®^ (Sigma-Aldrich, Diegem, Belgium) (a twentyfold dilution) for 30 min at room temperature in a glass Erlenmeyer on a shaker (Yellow Line OS10 shaker, IKA, Staufen, Germany) at 200 rpm. Maceration and washing liquids were filtered over a cellulose filter paper (595½ folded filters, 125 mm diameter, Whatman, VWR Leuven, Belgium) to remove plant debris and then over two 0.22 µm mixed cellulose ester filters (Millipore, Molsheim, France) to concentrate the bacteria from the large volumes (200 to 600 mL) to two small sample (two 0.22 µm filter discs) suitable for DNA extraction.

#### 2.2.2. DNA Extraction

Two protocols for extraction of the total microbial DNA were evaluated, namely the FastPrep^®^ (MP Biomedicals, Santa Ana, CA, USA) and the NucliSENS^®^ easyMAG^®^ (BioMérieux, Marcy l'Etoile, France) methods. These were applied to the different sample preparations, namely 300 mg leave samples and the 0.22 µm filters containing bacteria from the maceration or washing liquid. The FastPrep^®^ procedure consisted of addition of 200 mg glass beads (0.10 to 0.11 mm diameter, Sartorius, Goettingen, Germany) and 1 mL lysis buffer (containing 100 mM Tris, 100 mM EDTA, 100 mM NaCl, 1 % polyvinylpyrrolidone, 2 % sodium dodecyl sulphate), followed by mechanical lysis of the samples in the FastPrep^®^-96 Instrument (MP Biomedicals) by two cycles of 40 s at 1600 rpm. Next, the total nucleic acids were extracted with phenol:chloroform (Sigma-Aldrich), precipitated with sodium acetate (3 M) and isopropyl alcohol at −20 °C for 2 h and dissolved in 50 µL PCR-grade water (Sigma-Aldrich). The NucliSENS^®^ easyMAG^®^ (BioMérieux) generic protocol 2.0.1. was performed according to the instructions of the manufacturer, with on-board lysis (2 mL) and final elution in 25 μL. 

#### 2.2.3. PCR Amplification of 16S rRNA Gene

For DGGE, approx. 200 bp of the 16S rRNA gene containing the hypervariable region V3 was amplified using primers 338f-GC and 518r (5’-CGCCCGCCGCGCGCGGCGGGCGGGGCGGGGG CACGGGGGGACTCCTACGGGAGGCAGCAG-3’and 5’-Attaccgcggctgctgg-3’) [15] with 1 µL template in 25 µL reaction volumes containing 0.2 µM of each primer, 0.6 U recombinant *Taq* DNA polymerase (Thermo Scientific, St Leon-Rot, Germany), 1X Taq buffer with 5 mM KCl and 0.1 mM MgCl_2_, 200 µM dNTPs (dNTP Mix, Thermo Scientific) and 1.2 µg BSA (Bovine Serum Albumin, Roche, Mannheim, Germany) and the following temperature profile: 94 °C for 5 min and 30 cycles of 95 °C for 1 min, 53 °C for 1 min and 72 °C for 2 min, and finally 10 min at 72 °C. 

For NGS, approx. 500 bp spanning the V1, V2 and V3 region of the 16S rRNA gene was amplified with primers 27F and 533R (5'-AGAGTTTGATCCTGGCTCAG-3' and 5'-TTACCGCGGCTGCTG GCAC-3') [27,28] with 4 µL template in 100 µL reaction volumes containing 0.2 µM of each primer, 0.6 U recombinant *Taq* DNA polymerase (Thermo Scientific), 1× Taq buffer with 5 mM KCl and 0.1 mM MgCl_2_, 200 µM dNTPs (dNTP Mix, Thermo Scientific) and 1.2 µg BSA (Bovine Serum Albumin, Roche) and the following temperature profile: 94°C for 5 min and 30 cycles of 95 °C for 1 min, 57 °C for 1 min and 72 °C for 2 min, and finally 10 min at 72 °C. The primers were labeled at the 5’ with different multiplex identifiers for the different samples to allow multiplexing of samples for NGS.

#### 2.2.4. Denaturing Gradient Gel Electrophoresis (DGGE) 

The PCR fragments were separated on a 45 to 60 % (100 % denaturant comprised 7 M urea and 40 % formamide) DGGE gel containing 8 % (w/v) polyacrylamide gels in 1× TAE buffer run for 16 h at 60 °C at 38 V using the DCode system (BioRad, Temse, Belgium) [29]. Negative controls (no template controls) from the PCR were also run as negative controls for DGGE. Positive controls (markers) were at least once per six samples and consisted of an in-house bacterial mixture which yielded a complex band pattern spanning the full DGGE gel. After staining with SyberGreen I (Invitrogen, Life Technologies, Gent, Belgium), photos of the gels were analyzed using BioNumerics (Applied Maths, Sint-Martens-Latem, Belgium). The different lanes were defined, the background was subtracted, the intensity of the lanes was normalized and clustering was performed with the Pearson correlation of the pairwise similarities and the Ward dendrogram type. No attempt was made at identification based on the shorter (approx. 200 bp) DGGE amplicons of the 16S rRNA gene, but instead next-generation sequencing (NGS) of 500 bp 16S rRNA gene fragments of the mixed bacterial community on basil leaves was performed. 

#### 2.2.5. Next-Generation Sequencing (NGS) 

The PCR fragments were purified with the PureLink^®^ PCR Purification Kit (Life Technologies, Gent, Belgium). After measuring the DNA concentration the Qubit^®^ dsDNA HS Assay (Life Technologies), an equimolar mixture was subjected pyrosequencing on the Genome Sequencer (GS) FLX Titanium 454 System (Roche) at Beckman Coulter Genomics USA (Danvers, MA, USA). Bioinformatic analysis of the sequences was performed by Beckman Coulter Genomics France (Grenoble, France). Briefly, the sequences were de-multiplexed by sorting and removing of the barcodes. The MIRA v3.2 assembler (http://www.chevreux.org/projects_mira.html, available at http://sourceforge.net/projects/mira-assembler/files/MIRA/) was used in est-mode to cluster quality checked sequences. The resulting contigs and singletons (orphans) were blasted against an in-house curated copy of the Ribosomal Database Project (RDP) database v10.29 with only non-redundant sequences of sufficiently detailed and reliable phylogenetic annotation. Taxonomic classification and counting of the blast (http://blast.ncbi.nlm.nih.gov/Blast.cgi?CMD=Web&PAGE_TYPE=BlastHome) results was performed with Metagenome Analyzer (MEGAN4) on the 25 best hits (available at http://ab.inf.uni-tuebingen.de/software/megan4/). Dissimilarities in bacterial composition of the different samples was visualized using Principal Component Analysis (PCA) by the prcomp function in the R statistical software v3.2.1. All 16S rRNA sequence reads from this study are available from the Sequence Read Archive (SRA) under BioProject accession number PRJNA288639 on the NCBI website (http://www.ncbi.nlm.nih.gov/sra).

### 2.3. Culture-Based Microbiological Analyses

The cultivable fractions of the bacterial community on basil leaves was assessed after a standard non-selective enrichment step of approx. 25 g basil leaves in a tenfold larger volume of buffered peptone water (BPW, Oxoid, Erembodegen, Belgium) at 37 °C by taking samples of the BPW enrichment broth after various time points (5 h, 28 h, 30 h and 72 h). Which epiphytic basil bacteria are capable of growing on non-selective plates of tryptic soy agar (TSA, Oxoid), selective plates violet red bile lactose agar (VRBL, Oxoid) for coliforms and selective plates xylose lysine desoxycholate agar (XLD, Oxoid) for *Salmonella* was also assessed. Approx. 25 g basil leaves was tenfold diluted in physiological peptone salt (PPS) solution (containing 8.5 g/L NaCl (Fluka, Sigma-Aldrich, Diegem, Belgium) and 1 g/L neutralized bacteriological peptone (Oxoid)) and macerated in a stomacher for 1 min. Hundred µL of the appropriate tenfold dilution was plated on TSA, VRBL and XLD plates and these plates were incubated at 37 °C for 24 h. The microbial mass was washed off the plates with 1.3 mL sterile PPS and subjected to DNA extraction and further molecular microbiological analyses (see Section 2.2—Molecular microbiological analyses). 

### 2.4. Storage Experiments

Basil leaves were stored in 30 g portions in plastic bags at 7 °C, 15 °C and 22 °C. At each time point, two samples of 30 g basil leaves were analyzed, one of imported basil leaves from Israel (batch V) and one of basil leaves removed from Belgian whole plants (batch IV). Basil leaves were packaged in sealed bags of 15 cm × 15 cm with high permeability (4600 mL O_2_ per m² day at 7 °C, Amcor Flexibles, Gent, Belgium) with normal atmospheric conditions (*i.e.*, with air as initial headspace). The visual quality of basil leaves was assessed by the person (always the same person) performing the microbial analysis at each time point by scoring the general appearance, cold damage, decay, clean cutting, bruising, yellowing and blackening of the growth points on a scale of 1 to 5. An overall score was then calculated from these individual scores on a scale of 1 to 9 for overall visual quality. Scores of 5 or below correspond with spoiled basil samples, while scores of 6 or above are acceptable for consumption. The total microbial count was followed by plating on TSA (see Section 2.3. classical microbiological analyses) and by DGGE and NGS (see Section 2.2. molecular microbiological analyses). For the storage experiment, washing was selected as the sample preparation method because it resulted in higher diversity in the DGGE pattern and lower amounts of eukaryotic DNA. EasyMAG^®^ extraction was selected over FastPrep^®^ as the DNA extraction method because it generally resulted in slightly lower DNA yield but with higher purity than FastPrep^®^.

## 3. Results 

### 3.1. Culture-Independent Characterisation of the Bacterial Community

**Figure 2:**
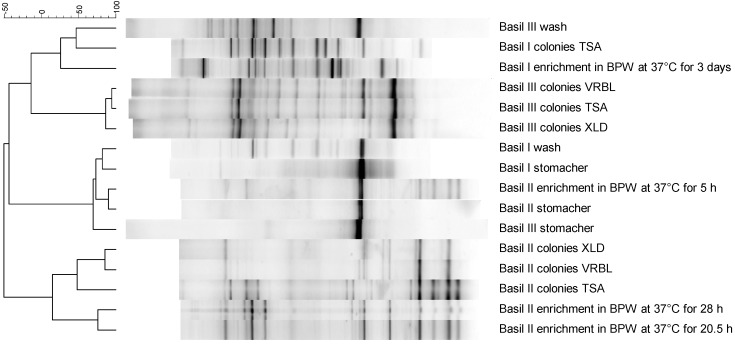
Denaturing gradient gel electrophoresis (DGGE) patterns of bacterial communities of basil batches I, II and III with different sample preparation methods and with and without cultivation steps.

Direct extraction of basil leaves failed to produce sufficient microbial DNA of sufficient purity for PCR analysis. Maceration of basil leaves in a stomacher, which constitutes the standard sample preparation prior to conventional microbiological analysis by plating or enrichment, showed one dominant band on DGGE (Figure 2) and one dominant bacterial genus, *Novosphingobium* spp., by NGS (Table 1) for all three basil batches. 

Washing of basil leaves resulted in DGGE patterns with more bands in addition to the dominant one, provided Tween 80^®^ was added. Washing of basil leaves in PPS without Tween 80® resulted in the same DGGE pattern (one dominant band at the same location) as maceration of basil leaves in a stomacher (results not shown).In accordance, more diverse results for the epiphytic bacteria (decreased dominance of *Novosphingobium* spp. and increased detection of *Pseudomonas* spp., Enterobacteriaceae, *Flavobacterium* spp. and *Sphingobacterium* spp.) were obtained by NGS after washing in comparison with maceration in a stomacher (Table 1). 

**Table 1:** Culture-independent identification of the bacterial communities on basil leaves from batch I, II and III by next-generation sequencing (NGS) of the 16S rRNA gene, showing bacterial groups and genera which constituted at least 1 % of the total bacteria (rescaled to 100 %).

Sample	Basil I, Stomacher	Basil I, Wash	Basil II, Stomacher	Basil II, Wash	Basil III, Stomacher	Basil III, Wash
Total number of reads	36,887	40,630	33,794	Failed	64,772	10,772
Median length of reads (bp)	473	474	473		473	473
Not assigned	29	694	51		20	6
Eukaryota	33,809	22,490	28,242		63,837	10,202
Bacteria (rescaled to 100% below)	3,050	17,446	5,501		915	564
Bacteroidetes		1%			1%	74%
*Arcicella*						1%
*Chryseobacterium*					1%	5%
*Flavobacterium*						11%
*Sphingobacterium*						56%
Alphaproteobacteria	87%	5%	83%		71%	17%
*Altererythrobacter*						2%
*Novosphingobium*	86%	4%	81%		71%	9%
*Sphingobium*						1%
*Sphingomonas*			1%			2%
Betaproteobacteria	1%	6%			1%	
*Herbaspirillum*		4%				
Gammaproteobacteria	10%	88%	16%		26%	5%
*Acinetobacter*		4%			18%	5%
*Pseudomonas*	1%	40%	4%		5%	
*Rheinheimera*					1%	
Enterobacteriaceae	8%	43%	11%		2%	
*Enterobacter*		15%	2%			
*Erwinia*	1%		2%			
*Klebsiella*	3%	11%	1%			
*Kluyvera*		1%				
*Pantoea*		6%	6%			
*Rahnella*		4%				
*Raoultella*	2%	2%				
Unclassified	1%	1%			1%	2%

The influence of the DNA extraction method seemed rather limited, but EasyMAG^®^ extraction generally resulted in lower DNA yield but DNA with higher purity (results not shown). Moreover, FastPrep^®^ once failed to extract sufficient DNA from multiple washing samples of basil batch II (so no results are available for this sample), so from thereon preference was given to the EasyMAG^®^ method. The elution volume of the extracted DNA, *i.e.*, the dilution degree of the template during PCR amplification, did not of affect the final DGGE result (results not shown). 

**Figure 3:**
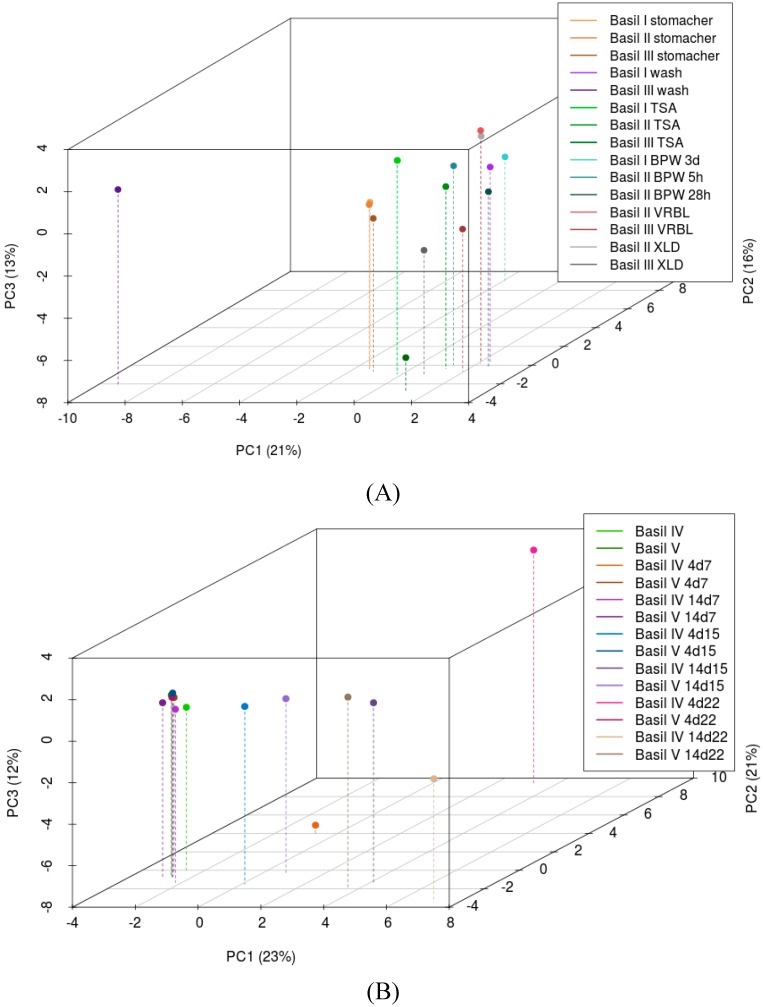
Principal component analysis of the NGS data of (**A**) basil batches I, II and III with different sample preparation methods and (**B**) storage of basil batches IV and V at different temperatures.

Filtration over paper filters to remove plant material and filtration of washing and maceration solutions over a 0.2 µm filter to up-concentrate bacterial densities prior to DNA extraction showed no influence on the DGGE pattern (results not shown), so both filtrations were routinely applied. Unfortunately but not unexpectedly, the main constituents of the basil samples were eukaryotic plant DNA fragments, ranging from 55 % to 99 % (Table 1). As expected, the type of sample preparation (maceration *vs.* washing) influenced the fraction of eukaryotic DNA, with the more gentle procedure of washing the leaves resulting in a relative increase of microbial DNA of 5.2-fold (basil batch I) and 3.7-fold (basil batch III).In accordance with DGGE clustering, principal component analysis (PCA) of the NGS data showed that the bacterial communities of basil batches I, II and III were very similar following the maceration sample preparation method with a stomacher (Figure 3A). After washing, the samples showed increased variability with basil III being an outlier. Samples derived by washing and by various culture steps from the same basil batch had the tendency to cluster together but were also intermixed with those of other batches, so no clear separation could be made with PCA.

### 3.2. Culturing of the Bacterial Community 

The dominant band in the DGGE pattern disappeared during enrichment (Figure 2). After 5 h enrichment in BPW at 37 °C, the similarity of the bacterial community with those of the original non-enriched basil samples was still large enough to cluster with these samples on DGGE. In accordance, NGS revealed that *Novosphingobium* spp. and eukaryotic plant DNA were still present after 5 h enrichment of basil II, but both disappeared during further enrichment (Table 2). Interestingly, all culture-derived samples from a specific basil batch clustered together during DGGE analysis, irrespective of being enriched in liquid medium or plated directly on different solid media (Figure 2). Enrichment of basil leaves in BPW at 37 °C resulted predominantly in *Bacteroides* spp. (42 %) and Enterobacteriaceae (34 %) for basil I and Enterobacteriaceae (55 %) and *Pseudomonas* spp. (37 %) for basil II (Table 2). Cultivation of the bacteria present on basil leaves on TSA, VRBL and XLD plates showed the growth of mainly Enterobacteriaceae, *Acinetobacter* spp., *Aeromonas* spp. and *Pseudomonas* spp. 

### 3.3. The Bacterial Community on Basil Leaves throughout Storage and Spoilage

**Figure 4:**
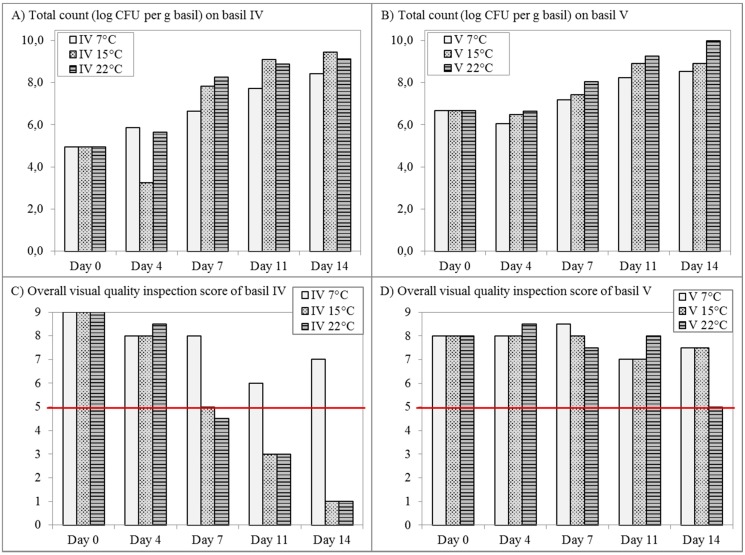
Changes in the total bacterial density on basil leaves from batch IV (**A**) and batch V (**B**) stored in bags at 7 °C, 15 °C and 22 °C for 14 days, determined by plating on TSA and assessment of the overall visual quality of basil leaves from batch IV (**C**) and batch V (**D**). Perfect quality corresponds with a score of 9, while the limit of acceptability for consumption lies at score 5 (indicated by the horizontal line), so all scores equal to or below 5 correspond with spoiled basil samples.

Changes in the bacterial community on cut and packages basil leaves was followed during 14 days of storage at different temperatures: 7 °C, 15 °C and 22 °C. In contrast to most fresh herbs, basil is sensitive to chilling injury, which means that storage at low temperatures (≤ 10 °C) causes brown discoloration, wilting of the leaves and loss of aroma [30]. The optimal storage temperature for basil is 15 °C, but storage at 7 °C and 22 °C was also investigated because many consumers store basil in their domestic refrigerator or at room temperature on a kitchen shelf. The total mesophilic count increased significantly during storage until very high levels of 8 to 10 log CFU/g were reached (Figure 4). The visual quality deteriorated from perfect (score 9) to no longer acceptable for consumption (score 5 or below). Large variation was observed between the two different batches in terms of spoilage. Basil IV was already spoiled after 7 days storage at 15 °C and 22 °C, but storage at 7 °C was possible for 14 days while remaining of acceptable quality for consumption. In contrast, basil leaves from batch V were only spoiled after 14 days at 22 °C and remained acceptable for consumption for 14 days at both 7 °C and 15 °C.

**Table 2:** Identification of bacteria from the basil communities I, II and III growing during BPW enrichments at 37 °C and on TSA, XLD and VRBL plates by next-generation sequencing (NGS) of the 16S rRNA gene, showing bacterial groups and genera which constituted at least 1 % of the total bacteria (rescaled to 100 %).

Sample	Basil I, BPW 3d	Basil II, BPW 5h	Basil II, BPW 28h	Basil I, TSA	Basil II, TSA	Basil III, TSA	Basil II, VRBL	Basil III, VRBL	Basil II, XLD	Basil III, XLD
Total number of reads	42,703	29,757	36,870	13,533	47,182	39,389	23,565	38,841	40,201	31,964
Median length of reads (bp)	509	494	500	503	502	496	500	496	499	503
Not assigned	1,829	1,985	2,294	299	3,839	378	114	712	226	198
Eukaryota	0	14,201	0	0	0	0	0	0	0	0
Bacteria (rescaled to 100% below)	40,874	13,572	34,576	13,234	43,343	39,011	23,451	38,129	39,975	31,766
Actinobacteria				12%						
*Arthrobacter*				8%						
*Kocuria*				2%						
Bacteroidetes	55%			4%						
*Bacteroides*	42%									
*Macellibacteroides*	3%									
*Parabacteroides*	11%									
*Chryseobacterium*				3%						
Clostridia	6%		1%							
*Clostridium*	5%									
Alphaproteobacteria		5%								
*Novosphingobium*		5%								
Betaproteobacteria			6%			3%		2%		
*Comamonas*			5%			2%		2%		
Gammaproteobacteria	38%	95%	93%	83%	100%	97%	100%	98%	100%	100%
*Aeromonas*						38%		31%		36%
*Alishewanella*						6%				
*Shewanella*								1%		1%
*Rheinheimera*						13%				
*Acinetobacter*	4%		1%	45%		17%		6%		
*Pseudomonas*		1%	37%	14%	16%	13%	6%	23%	1%	55%
*Stenotrophomonas*				1%			1%			
Enterobacteriaceae	34%	94%	55%	23%	84%	10%	92%	36%	99%	7%
*Aranicola*								2%		
*Cedecea*			2%							
*Citrobacter*	17%		1%				1%		2%	
*Enterobacter*	8%	10%	15%	5%	5%	1%	11%	16%	11%	3%
*Erwinia*		10%	5%	6%	13%		28%		28%	
*Klebsiella*	3%	1%	7%	4%			5%	7%	6%	
*Kluyvera*		1%	1%	1%	1%		4%		6%	
*Pantoea*	2%	70%	21%	3%	61%	1%	41%	7%	45%	1%
*Pectobacterium*					3%	7%				
*Raoultella*	1%			2%						

**Figure 5:**
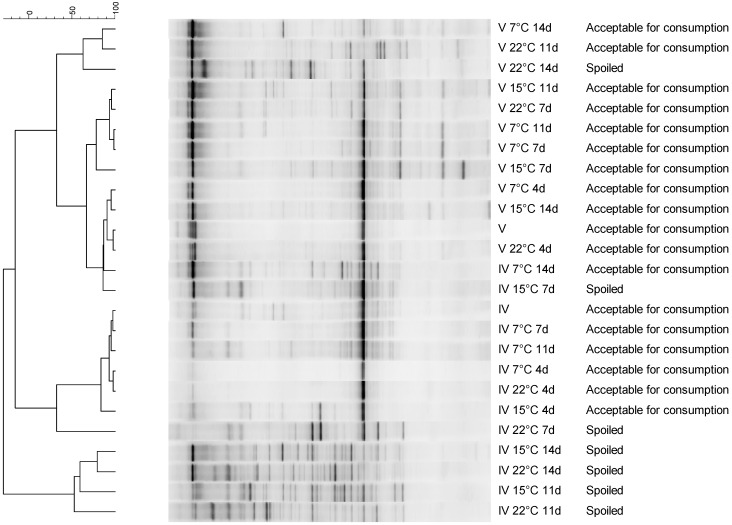
Denaturing gradient gel electrophoresis (DGGE) patterns of bacterial communities on basil leaves from batch IV and batch V stored at 7 °C, 15 °C and 22 °C for 14 days.

DGGE patterns of basil samples clustered into one heavily spoiled group of basil IV and a group of non-spoiled or at the limit of spoilage with score 5 (Figure 5). The latter was in turn divided into a group of basil IV samples and basil V, with the only exception of two basil IV samples (stored 14 days at 7 °C and 7 days at 15 °C), which were more similar to the other basil batch. Spoilage is thus associated with numerical increases and compositional changes in the bacterial community detectable by DGGE. However, the colonies from the total plate counts were also subjected to DGGE analysis, but no meaningful clustering was observed (data not shown). 

NGS sequencing showed that storage of basil leaves from both basil batches IV and V at non-refrigerator temperatures (15 °C and 22 °C) resulted in an increase of Bacteriodetes (mainly *Chryseobacterium, Flavobacterium, Pedobacter* and *Sphingobacterium* species) from <1 % of the population to between 14% and 20% (Table 3). *Pseudomonas* spp. were always present on cut and packed basil leaves, but prolonged storage (14 days) at room temperatures (15 °C and 22 °C) decreased their proportion, while it increased at refrigeration temperature (7 °C). Enterobacteriaceae proportions remained stable or increased during storage. In particular *Enterobacter* and *Rahnella* species grew out during storage of basil IV and *Enterobacter* and *Pantoea* species on basil V. The observed shifts in the bacterial community were variable, more in relation to the storage temperature and to the composition of the initial bacterial community than to spoilage. Despite strong bacterial outgrowth during storage, communities of basil batches V were very similar by PCA (Figure 3B). Basil IV showed higher variability in the composition of the bacterial community and increased spoilage rates.

**Table 3:** Identification of bacteria from the basil communities IV and V before, after 4 days and after 14 days of storage at 7 °C, 15 °C and 22 °C by next-generation sequencing (NGS) of the 16S rRNA gene, showing bacterial groups and genera which constituted at least 1 % of the total bacteria (rescaled to 100 %).

Sample	Basil IV Day0	Basil IV 7d 4°C	Basil IV 7d 14°C	Basil IV 15d 4°C	Basil IV 15d 14°C	Basil IV 22d 4°C	Basil IV 22d 14°C	Basil V Day0	Basil V 7d 4°C	Basil V 7d 14°C	Basil V 15d 4°C	Basil V 15d 14°C	Basil V 22d 4°C	Basil V 22d 14°C
Total number of reads	36.746	35.279	63.993	11.892	24.615	49.852	49.347	60.524	57.824	83.574	37.559	51.080	70.829	64.761
Median length of reads (bp)	438	456	404	437	405	464	393	417	459	402	437	457	453	393
Not assigned	56	40	354	109	788	32	700	94	90	928	181	151	208	3.815
Eukaryota	35.626	34.556	26.747	9.870	51	49.363	222	46.475	53.834	1.735	27.767	48.859	56.755	9.132
Bacteria (rescaled to 100 % below)	1.065	682	36.892	1.913	23.776	457	48.425	13.954	3.900	80.911	9.611	2.070	13.867	51.815
Actinobacteria		14%	1%	1%		14%						1%		
*Microbacterium*						2%						1%		
*Arthrobacter*		10%		1%		8%								
Bacteroidetes		4%	2%	17%	29%	18%	14%					16%		19%
*Chryseobacterium*		2%			1%		11%					3%		1%
*Flavobacterium*			2%	17%	25%		1%					3%		
*Pedobacter*					2%	18%	2%							
*Sphingobacterium*												9%		18%
Bacilli						2%	1%							
*Bacillus*						2%								
Clostridia							3%							
*Clostridium*							2%							
Alphaproteobacteria	5%	20%		8%	11%	21%	8%	2%	8%		2%	23%	4%	8%
*Methylobacterium*						9%					1%			
*Agrobacterium*					1%		1%					8%		1%
*Rhizobium*				2%	2%		1%					2%		2%
*Novosphingobium*	5%	19%		4%	6%	8%	3%	2%	7%		1%	11%	3%	3%
*Sphingomonas*				1%	1%		1%					1%		1%
Betaproteobacteria	28%	15%	2%	9%	15%	36%	20%					6%		28%
*Achromobacter*							3%					1%		1%
*Burkholderia*				2%			1%					1%		
*Acidovorax*	5%													
*Comamonas*	1%	1%												
*Delftia*							1%							8%
*Variovorax*						2%								
*Duganella*	1%				3%									
*Herbaspirillum*		5%			2%		6%							
*Herminiimonas*							1%							
*Janthinobacterium*	17%		1%	2%								2%		
*Oxalicibacterium*					1%		2%							5%
*Methylobacillus*														12%
*Methylophilus*				2%	4%	32%	4%							
*Zoogloea*					2%									
Gammaproteobacteria	66%	44%	94%	64%	44%	7%	54%	97%	91%	100%	97%	54%	95%	45%
*Pseudoalteromonas*		3%								1%				
*Acinetobacter*		4%												
*Pseudomonas*	56%	30%	71%	42%	10%	3%	22%	96%	85%	95%	95%	44%	89%	24%
*Luteibacter*				1%	6%		1%							
*Stenotrophomonas*	1%				22%	2%	17%					4%		12%
*Xanthomonas*					1%		2%							1%
Enterobacteriaceae	8%	7%	22%	21%	5%		10%	1%	6%	3%	2%	5%	5%	8%
*Buchnera*		2%												
*Enterobacter*			3%	3%	2%		7%						1%	6%
*Erwinia*	3%													
*Ewingella*			4%	11%										
*Pantoea*	4%				1%			1%	5%	2%	1%	5%	3%	1%
*Rahnella*		4%	10%	5%	1%		1%							
*Serratia*			5%	1%										
Unclassified		2%												

## 4. Discussion 

This study is the first to investigate the total bacterial community on fresh basil leaves without prior cultivation steps and identified *Novosphingobium* spp. as the dominant bacterial genus on basil leaves. *Novosphingobium* spp. have mostly been isolated from soil [31] and various aquatic environments, including winery wastewater [32], pulp and paper factory wastewater [33], lake water [34] and a sewage pond [35]. Some species of *Novosphingobium* can grow at the standard incubation temperatures 30 °C and 37 °C [33], but most others have a more psychrotrophic nature and do not grow at temperatures ≥30 °C [36]. The most prominent characteristics of *Novosphingobium* spp. are the production of exopolysaccharides (EPS) [31], the reduction of nitrate [35,36], the fixation of nitrogen [33], the degradation of lignin and cellulose [37,38], and the metabolism of aromatic compounds [34,36]. The ability to fix nitrogen is a beneficial characteristic, which significantly promotes the plant’s growth [39]. Degradation of aromatic compounds is highly interesting for bioremediation applications [36]. Moreover, basil contains several antimicrobial aromatic compounds such as estragole (12%) and methyl cinnamate (7%), which inhibit or kill a wide range of bacteria [40]. Resistance to and utilization of these essential oils would provide an obvious advantage to *Novosphingobium* spp. to colonize basil leaves and maintain itself as a dominant member of the epiphytic bacterial community. A similar phenomenon has been observed for pathogenic bacteria. *Salmonella* Senftenberg from a foodborne outbreak with packed fresh basil [41] showed increased tolerance towards the antimicrobial compounds linalool, estragole and eugenol in basil oil, which also led to increased survival and persistence on basil [42]. The metabolic properties of *Novosphingobium* spp. isolated from basil thus constitute an interesting topic for further research to confirm or disprove this hypothesis. 

Dominant bacteria other than *Novosphingobium* spp. belonged primarily to Gammaproteobacteria, *i.e.*, *Acinetobacter, Pseudomonas* and Enterobacteriaceae (mainly *Enterobacter, Erwinia, Klebsiella, Pantoea*) and Bacteriodetes (mainly *Flavobacterium* and *Sphingobacterium* species). An overview of the characteristics of these dominant bacterial genera is given in Table 4. Most genera comprise species which are naturally occurring on plant leaves, or in the environment (soil, water) from which they can be transferred to leaves [1]. Most of the genera contain species which are opportunistic pathogens for humans. This means that occasionally infections occur, but typically in vulnerable persons with wounds or underlying illness. Such infections often taken place in a hospital setting (*i.e.*, nosocomial infections) and typically occur through wounds or medical devices such as catheters, not through ingestion of these bacteria with food. As such, consumption of fresh herbs does not present a health risk from the naturally occurring bacteria. The bacterial community naturally present on basil leaves may function as a barrier against long-term contamination with human pathogens from soil, as demonstrated by a study in which basil seedlings were contaminated with *Listeria monocytogenes* from soil but which were no longer contaminated as mature plants at harvest [43]. Nevertheless, herbs may become contaminated, e.g. via contaminated irrigation water, with foodborne pathogens such as *Salmonella* and pathogenic *E. coli* which may cause gastrointestinal disease, as illustrated by outbreaks with fresh basil or fresh basil pesto in 2006 [44], 2007 [45] and 2011 [46].

**Table 4:** Characteristics of the dominant bacterial genera detected on basil leaves [47].

Genus	Gram Staining	Respiratory Metabolism	Motility	Temperature Range Growth	Habitat	Pathogenicity
*Flavobacterium*	Gram negative	Aerobic	Nonmotile or motile by gliding	−7 to 45 °C	Soil, freshwater, marine and saline environments	Some species, such as *F. columnare*, *F. psychrophilum* and *F. branchiophilum*, are pathogenic for freshwater fish. Some strains of *F. johnsoniae* are plant pathogens causing soft rot in various plants.
*Sphingobacterium*	Gram negative	Aerobic	Sliding motility	2 to 45 °C	Soil and composted manure	Some species are opportunistic pathogens for humans.
*Acinetobacter*	Gram negative	Aerobic	Twitching motility by fimbriae	20 to 37 °C	Soil, water, sewage and plants	Although considered normally nonpathogenic, they may cause nosocomial infections such as bacteremia, secondary meningitis, pneumonia, and urinary tract infections in humans.
*Pseudomonas*	Gram negative	Aerobic	Motile by one or several polar flagella and fimbriae	4 to 45 °C	Plants (rhizospheres and leave surfaces) and soil	Some species are pathogenic for humans, animals, or plants. Plant pathogenic species such as *P. syringae* may cause tumorous outgrowth, rot, blight or chlorosis, and necrosis in plants due to secretion of substances (such as toxins, plant hormones and enzymes) which alter the normal metabolism of plant cells. Others are opportunistic pathogens for animals and humans, such as *P. aeruginosa*.
*Citrobacter*	Gram negative	Facultatively anaerobic	Usually motile by peritrichous flagella	5 to >37 °C	Intestinal tract of humans and some animals, soil, water, sewage, plants and food (vegetables, dairy, fish)	Some species are opportunistic pathogens for humans.
*Enterobacter*	Gram negative	Facultatively anaerobic	Motile by peritrichous flagella	4 to 44 °C	Plants (rhizophere and leaves) and the intestinal tract of humans and animals	Some species are plant pathogens, such as *E. nimipressuralis* (wetwood in elm trees), *E. cancerogenus* (canker disease of *Populus* species) and *E. pyrinus* (brown leaf spot disease in pears).
*Erwinia*	Gram negative	Facultatively anaerobic	Motile by peritrichous flagella	0 to 40 °C	Plants	Plant pathogens which cause mainly blights and wilts. Infection through natural openings and wounds, followed by spread through the vascular tissue.
*Klebsiella*	Gram negative	Facultatively anaerobic	Nonmotile (except *K. mobilis*)	5 to 45 °C	Intestinal tract of humans and animals, soil, water, sewage and plants	Opportunistic and nosocomial human pathogens, e.g. *K. pneumoniae*, causing pneumonia, urinary tract infections, bacteremia and sepsis.
*Kluyvera*	Gram negative	Facultatively anaerobic	Motile	4 to 40 °C	Intestinal tract of humans and animals, soil, sewage and food (milk, dairy and other food products of animal origin)	Opportunistic human pathogen.
*Pantoea*	Gram negative	Facultatively anaerobic	Most strains are motile by peritrichous flagella	4 to 41 °C	Plants, seeds, fruits, soil and water	Some strains are opportunistic pathogens for plants, humans and animals.
*Rahnella*	Gram negative	Facultatively anaerobic	Motile by peritrichous flagella	1 to ≥37 °C	Fresh water, soil, plant rhizosphere, intestinal tract of snails	Opportunistic human pathogens causing wound infections, bacteremias, acute gastroenteritis and septicemia.

Standard microbiological analyses are performed at high incubation temperatures, typically 37 °C, because these analyses are aimed at retrieving medically important bacteria. In contrast, many environmental bacteria, including the *Novosphingobium* spp. in our study, cannot grow (fast enough) during such cultivation steps and thus disappear from the bacterial community. In accordance, a previous study reported mainly *Enterobacter* species on basil leaves by NGS sequencing, but after an overnight enrichment step in brain heart infusion (BHI) broth at 37 °C [48]. The natural occurrence of Enterobacteriaceae, and more specifically the thermotolerant “faecal” coliforms such as *Enterobacter* spp., *Klebsiella* spp. and *Citrobacter* spp., on basil leaves precludes the use of these bacterial groups to assess the microbiological quality and safety of vegetable food products including fresh herbs, since these are not unequivocally linked to faecal contamination and thus the presence of human pathogens [49]. 

Spoilage of packed cut basil leaves was associated with bacterial growth exceeding 7 log CFU/g during storage at 15 °C and 22 °C, but not consistently. Sensorial quality of basil leaves was primarily impacted by physiological tissue deterioration and visual defects such as discoloration, dehydration and curling. Thus, spoilage of basil leaves could not be attributed to the growth of specific bacterial genera. In contrast, the increase in bacterial numbers and diversity was more likely the consequence rather than the cause of spoilage due to the increased release of nutrients during the physiological degeneration of the basil leaves.

This study is another example of the well-known fact of how conventional culture-based techniques provide a biased and fractional view of the bacteria present, missing or severely underestimating the dominant bacteria initially present. The culturing bias can be avoided by application of molecular methods, but it is important to realize that all steps in the DGGE and NGS protocols also involve choices which are potentially associated with other biases. 

Sample preparation may exert a major influence on how many and which bacteria are sampled and analysed. In the present study, washing of basil leaves in a solution with addition of a detergent (Tween 80^®^) yielded numerically less bacteria (approximately tenfold lower counts) but a higher diversity of species than maceration. Tween 80® enhanced the removal of bacterial biofilms and the disaggregation of bacterial cell clumps, as previously confirmed for *Salmonella enterica* on cilantro leaves [50]. The lower relative frequencies of *Novosphingobium* spp. after washing in comparison with maceration are most likely the result of a stronger than average attachment to the basil leaves, presumably in biofilms [32,50,51]. The choice of DNA extraction method may affect the outcome due to different lysis efficiencies for different bacterial species and for cells and spores [52,53,54], although the effect was limited in this study and others (e.g., [55]). It may be challenging to argue which specific sample preparation method will lead to the “true” and correct results. Nevertheless, acknowledgement and further investigation of these differences remains important, because sometimes (as here in the present study) the results may be very different and may lead to different conclusions. 

Without a prior culturing step, large amounts of eukaryotic basil DNA were co-purified and co-amplified, so the coverage of bacterial sequences decreased strongly from on average 42,370 reads per sample (minimum 10,772 and maximum 83,574) to on average 21,887 reads per sample (minimum 457 and maximum 80,911). As a result, sub-dominant bacterial members may not have been detected in the macerated and washed basil samples. Nevertheless, this study showed the importance of *Novosphingobium* spp. as a universal and numerically abundant epiphytic bacterium on basil. The problem of amplification of eukaryotic DNA could be avoided by use of specific primers [56], but careful design is then required to ensure amplification of the 16S rRNA variations of all different bacterial phylogenetic groups [57]. Alternative to 16S rRNA gene sequence itself, the rRNA internal transcribed spacer (ITS) region may be used for sequencing [58]. PCR amplification bias occurs due to the choice of conserved regions for the primers. Mismatches or other exceptional variations in the primer sequences present in one or more lineages in the targeted 16S rRNA gene region may create a bias against taxonomical groups by preferential amplification of perfectly matched sequences [59]. This effect may be augmented by the addition of multiplex identifiers and/or sequencing adapters to the primers. The region and the length of the 16S rRNA gene fragment determines the taxonomic precision which can be obtained [60,61,62]. Unfortunately, there is no hypervariable region of the 16S rRNA gene which offers good taxonomic coverage for all bacterial genera, but the V1/V2 and the V2/V3 regions have been the region of choice to distinguish most bacterial species to the genus level [60,61], so in this study a fragment containing the V1, V2 and V3 region was selected for NGS and the V3 region was used for DGGE. 

Different technologies and platforms for NGS exist, all with their own specific benefits and drawbacks [62]. In this study, the preference was given to 454 pyrosequencing due to the low error rate [63] and the longer fragments (*i.e.*, the V1/V2/V3 region of approx. 500 bp) which could be sequenced to enhance the reliability of identification and taxonomic classification [64]. On the other hand, the coverage was lower, especially due to the amplification of plant chloroplast DNA, which precludes the detection of more rare community members and limits the conclusions of this study to the dominant bacteria. An important limitation of this study is that only biological replicates have been analysed, this precludes statistical diversity and similarity analyses which could be performed on technical replicates of each biological sample. Specifically for NGS, bioinformatics analysis may be another major source of variability, since the same raw data may yield different results due to different clustering, alignment and annotation methods and the use of different databases with reference species [61], although often the same biological conclusion can be drawn [65]. It is very important to use an extensive database with quality checked 16S rRNA genes to ensure the accuracy of taxonomic identification, such as for example curated copies of the Ribosomal Database Project (RDP) [66], GreenGenes [67] and SILVA [68].

## 5. Conclusions

*Novosphingobium* spp. was the dominant bacterial genus identified after maceration of basil leaves, while diverse Gammaproteobacteria were found after washing of leaves, demonstrating the large impact of sample preparation methods on the results of culture-independent analyses such as DGGE and NGS. *Flavobacterium* spp., *Sphingobacterium* spp., *Acinetobacter* spp., *Pseudomonas* spp., *Enterobacter* spp., *Erwinia* spp., *Klebsiella* spp. and *Pantoea* spp. were frequently retrieved from basil leaves. These genera are often associated with plants and/or their natural environment (soil and water). Although some species are opportunistic human pathogens, consumption of naturally occurring bacteria on basil leaves presents no risk of gastrointestinal illness. 
